# Off-label treatment with miltefosine for complex, pediatric Old World cutaneous leishmaniasis

**DOI:** 10.1016/j.jdcr.2025.05.054

**Published:** 2025-07-30

**Authors:** Marlene Buchelt, Teresa Valero, Tamar Kinaciyan, Julia Walochnik, Maximilian Egg, Anna Szelenyi, Sophie Langer, Alessandra Handisurya

**Affiliations:** a Department of Dermatology, Medical University of Vienna, Vienna, Austria; b Institute of Specific Prophylaxis and Tropical Medicine, Center for Pathophysiology, Infectiology and Immunology, Medical University of Vienna, Vienna, Austria; c Division of General and Paediatric Radiology, Department of Biomedical Imaging and Image-Guided Therapy, Medical University of Vienna, Vienna, Austria; d Department of Pediatric and Adolescent Surgery, Medical University of Vienna, Vienna, Austria

**Keywords:** child, cutaneous leishmaniasis, *Leishmania infantum/donovani* complex, miltefosine, Old World leishmaniasis, pediatric

## Introduction

Leishmaniasis is an infectious disease caused by a group of parasitic protists of more than 20 different species, transmitted through the bites of phlebotomine sandflies. It can be classified into either Old World or New World leishmaniasis, based on the geographical origin of the causative species, and into cutaneous, mucosal/mucocutaneous, or visceral leishmaniasis, according to the clinical presentation.

Worldwide, more than 1 million new cases of cutaneous leishmaniasis (CL) are estimated annually with rising incidences. Although Austria is nonendemic,^1^ a 2.7-fold increase of CL cases was reported in 2010-2019 as compared to 2000-2009.^2^ A substantial proportion affected children aged <12 years often originating from countries such as Afghanistan, Syria, and Tunisia, all endemic for CL.^1^^,^^2^ Notably, species belonging to the *Leishmania* (*L*.) *donovani/infantum* complex, which are typically associated with visceral leishmaniasis, were increasingly detected in the population as a causative agent.^3^

## Case report

**Fig 1:**
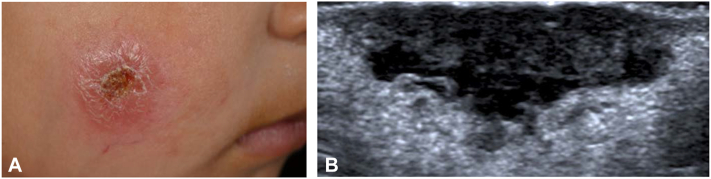
Cutaneous leishmaniasis lesion caused by a member of the *Leishmania donovani/infantum* complex on the right cheek of a 3-year-old girl. **A,** Ulcerated, erythematous plaque sized 3 cm in diameter with raised borders and central crusting. **B,** Ultrasound image showing a well-defined, inhomogeneous, hypoechoic subcutaneous lesion measuring 2 × 1 cm on the right cheek.

A 3-year-old girl of Syrian origin presented with an ulcerated plaque on her right cheek (Fig 1, *A* and *B*), which had slowly progressed from a papulonodular lesion within a year. The father reported onset during the family’s migration from Syria through Egypt and recalled multiple insect bites. In Egypt, her brother had been successfully treated for CL; however, the girl’s lesion had been (mis-)diagnosed as hemangioma and treated with laser. Based on the clinical appearance, the migratory route through endemic countries,^1^ and the history of insect bites and leishmaniasis in the sibling, CL was suspected and verified by multiplex real-time polymerase chain reaction–based detection of *L. donovani/infantum* complex DNA in fine needle aspirates obtained from the lesion’s borders. Blood analyses were negative for antileishmanial immunoglobulin G antibodies and *L.* DNA; lymph node sonography and physical examinations were inconspicuous.

**Fig 2:**
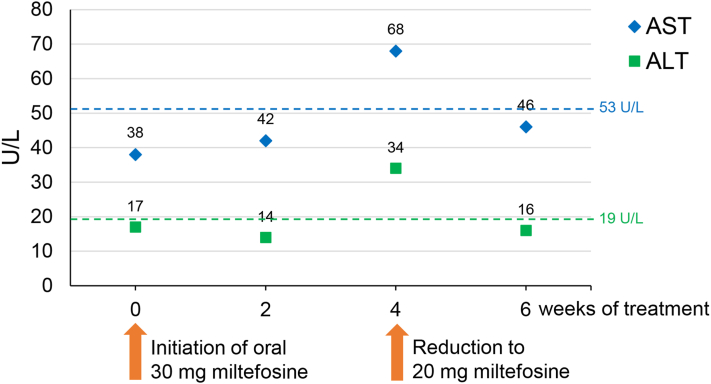
Levels of transaminase enzymes, alanine aminotransferase (ALT) (*green squares*) and aspartate transaminase (AST) (*blue diamonds*), over the course of treatment with miltefosine. The liver enzymes were determined prior to (week 0) and every second week during therapy. The time points of the treatment initiation with 30 mg miltefosine per day and of the dose reduction to 20 mg per day are depicted (by *orange arrows*). The *green* and *blue dotted lines* indicate the upper limits of the normal ranges of ALT and AST, respectively.

Given the complexity of the disease, ie, the facial localization and persistence for over a year, systemic treatment with miltefosine (Impavido) was initiated at a daily dose of approximately 2.5 mg per kg body weight, amounting to 30 mg. Treatment was well tolerated, albeit mild nausea and vomiting during the first few days were reported, which subsided immediately with concomitant intake of fatty food. Prior to and throughout treatment, blood count and chemistry were monitored (Fig 2). Four weeks after initiation of miltefosine, increasing liver enzyme levels of the child warranted dosage reduction to 20 mg once daily, which was continued until the end of therapy.

**Fig 3:**
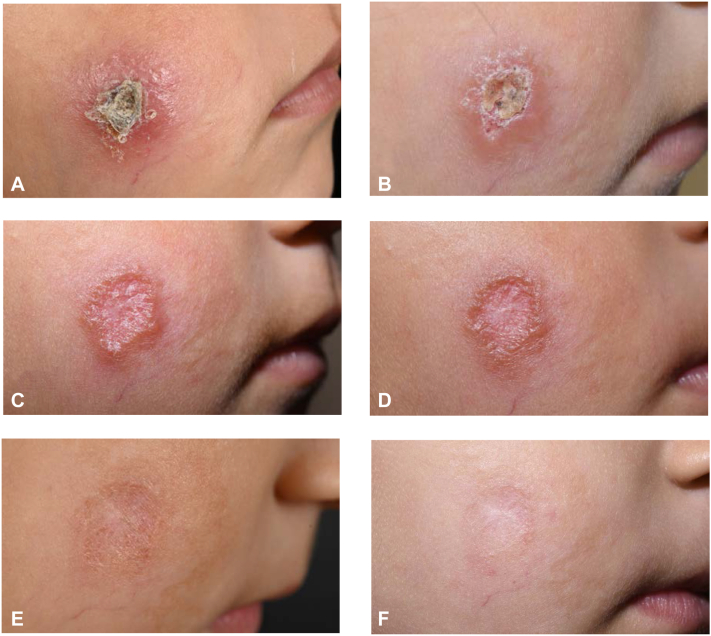
Clinical course of the lesion on a treatment with miltefosine. Lesion shown at **(A)** 2 weeks, **(B)** 4 weeks, **(C)** 6 weeks, and **(D)** 8 weeks after initiation of treatment. After 8 weeks of treatment, therapy with miltefosine was terminated. Residual atrophic scar around **(E)** 5 months and **(F)** 10 months after completion of therapy.

Prompted by miltefosine, healing of the lesion was noted over time (Fig 3, *A*-*D*). At week 8, the lesion had significantly improved (Fig 3, *D*), allowing for discontinuation of therapy. Follow-up visits revealed a residual atrophic scar with postinflammatory hyperpigmentation and absence of leishmanial DNA in the tissue (Fig 3, *E* and *F*).

## Discussion

CL is generally a self-limited condition that resolves within 3-18 months, with up to 10% of cases progressing to chronic disease. However, systemic treatment is recommended for complex forms of Old and New World CL to induce healing and avoid complications.^4^^,^^5^ Available treatment options include pentavalent antimonials, pentamidine isethionate, liposomal amphotericin B, azoles, and miltefosine.

The use of antimonials, which have longtime been regarded as first-line drugs for all leishmaniasis forms, has been hampered by their substantial toxicity profile, the emergence of drug resistance in several regions, and the potential reactivation of disease after clinical cure.^6^ Furthermore, some studies indicated lower efficacy in children compared to adults.^6^^,^^7^ Similarly, pentamidine is considered a less attractive option due to its potentially irreversible toxicity and variable efficacy against different *L.* species.^6^ Although liposomal amphotericin B has demonstrated high response rates, the substance’s nephrotoxicity, intravenous administration route, and high costs limit its use.^6^ Albeit azoles can be administered orally and have lower toxic profiles than antimonials, their efficacy has not been consistently proven for leishmaniasis.

Miltefosine, an alkylphosphocholine shown to be effective against a variety of *L.* species with cure rates between 68% and 85%, is currently approved for adults and children aged ≥12 years weighing ≥30 kg for the treatment of visceral leishmaniasis caused by *L. donovani*; CL caused by the New World species *L. braziliensis*, *L. guyanensis*, and *L. panamensis*; and mucosal leishmaniasis caused by *L. braziliensis*.^8^ Several studies showed its efficacy against visceral and cutaneous forms in the pediatric population under age 12.^9^, ^10^, ^11^, ^12^ Notably, the efficacy of miltefosine was higher or noninferior when compared to parenteral antimonials, specifically meglumine antimoniate.^9^, ^10^, ^11^ The oral mode of application facilitates treatment and miltefosine is usually well tolerated. The most common side effects include diarrhea, nausea, vomiting, and elevation of liver transaminases and serum creatinine. Currently, the drug’s costs and limited availability need to be considered, although these issues might change in near future since patent protection has expired.

Herein, the dose of 2.5 mg per kg body weight was aimed at, according to the World Health Organization recommendation for children aged 2-11 years with visceral leishmaniasis.^6^ However, dose adaptation had to be performed due to the predetermined formulation in capsules, each containing 10 mg of miltefosine. Treatment duration was prolonged due to the activity of the lesion’s borders until week 8, the lower steady-state concentrations in children, and to compensate for the lower dosage during weeks 4-8.^13^^,^^14^ We refrained from further extensions due to the elevated liver transaminases and reported detection of miltefosine in peripheral blood mononuclear cells and in plasma up to 1 and 6 months, respectively, after completion of treatment,^14^ indicating that the substance’s therapeutic effects may persist at the site of infection after discontinuation.

This case demonstrates that miltefosine can be used (1) in Old World CL, (2) is effective against CL caused by species of the *L. donovani/infantum* complex, and (3) is applicable to young children below the approved age.

Due to forced migratory movements and displacements, increasing international travel activities, and changes in vector ecology, CL poses an emerging public health issue, not only in regions with high prevalences of leishmaniasis but also in nonendemic regions. Hence, guidelines on the therapy of children with leishmaniasis are urgently needed. This task is admittedly challenging given the paucity of well-designed, randomized, controlled trials and the sparseness of comparative literature in both children and adults. To overcome the divergence in treatment procedures, durations of therapy, and definitions of outcome, procedural harmonization is warranted.

## Conflicts of interest

None disclosed.
